# Coarse- and fine-scale patterns of distribution and habitat selection places an Amazonian floodplain curassow in double jeopardy

**DOI:** 10.7717/peerj.4617

**Published:** 2018-05-16

**Authors:** Gabriel A. Leite, Izeni P. Farias, André L. S. Gonçalves, Joseph E. Hawes, Carlos A. Peres

**Affiliations:** 1 Programa de Pós-Graduação em Genética, Conservação e Biologia Evolutiva, Instituto Nacional de Pesquisas da Amazônia, Manaus, Amazonas, Brazil; 2 Laboratório de Evolucão e Genética Animal, Departamento de Genética, Universidade Federal do Amazonas, Manaus, Amazonas, Brazil; 3 Centre for Ecology, Evolution and Conservation, School of Environmental Sciences, University of East Anglia, Norwich, UK; 4 Grupo de Pesquisas em Mamíferos Amazônicos (GPMA), Instituto Nacional de Pesquisas da Amazônia, Manaus, Amazonas, Brazil; 5 Applied Ecology Research Group, Department of Biology, Anglia Ruskin University, Cambridge, UK

**Keywords:** Conservation, Cracids, Ecology, Várzea, Floodplain forest, Telemetry, Brazil

## Abstract

Patterns of habitat selection are influenced by local productivity, resource availability, and predation risk. Species have taken millions of years to hone the macro- and micro-habitats they occupy, but these may now overlap with contemporary human threats within natural species ranges. Wattled Curassow (*Crax globulosa*), an endemic galliform species of the western Amazon, is threatened by both hunting and habitat loss, and is restricted to white-water floodplain forests of major Amazonian rivers. In this study conducted along the Juruá River, Amazonas, Brazil, we quantified the ranging ecology and fine-scale patterns of habitat selection of the species. We estimated the home range size of *C. globulosa* using conventional VHF telemetry. To estimate patterns of habitat selection, we used geo-locations of day ranges to examine the extent and intensity of use across the floodplain, which were then compared to a high-resolution flood map of the study area. We captured two females and one male, which we monitored for 13 months between September 2014 and September 2015. Average home range size was 283 ha, based on the 95% aLoCoH estimator. Wattled Curassows selected areas of prolonged flood pulses (six to eight months/year) and had a consistent tendency to be near open water, usually in close proximity to river banks and lakes, especially during the dry season. Amazonian floodplains are densely settled, and the small portions of floodplain habitat used by Wattled Curassows are both the most accessible to hunters and most vulnerable to deforestation. As a result, the geographic and ecological distribution of Wattled Curassows places them at much higher extinction risk at multiple spatial scales, highlighting the need to consider habitat preferences within their conservation strategy.

## Introduction

Habitat availability, movement patterns and habitat use are driven by the abundance, availability, and distribution of resources, as well as by the landscape structure in which they are distributed (e.g., habitat patches and connectivity between them) (Beyer et al., 2010). Understanding how animals establish and use their home ranges within landscapes in order to meet their resource requirements (e.g., food, water, and breeding habitat) is vital to determine their potential vulnerability to extinction and to successfully design subsequent wildlife management programmes (Morrison, Marcot & Mannan, 1998; Willems & Hill, 2009). Recording movement patterns and habitat use is particularly crucial for threatened taxa. However, this information is often most deficient when populations occur at low densities or are difficult to observe due to evasive behaviour (Rechetelo et al., 2016), or sheer remoteness and inaccessibility of habitat.

It is important to incorporate habitat selection studies into conservation planning (Caughley, 1994), including assessments of both demographic and geographic definitions of rarity (Williams et al., 2009). Demographically rare species are typically expected to find some relief from direct anthropogenic threats, such as hunting, as a result of their low densities, unless they represent a particularly valuable commodity (e.g., ivory) whose value may rise with increased rarity (McClenachan, Cooper & Dulvy, 2016). Geographic rarity, linked to habitat specialisation, is also important in determining a species’ ability to persist in human-disturbed landscapes (Vergara & Armesto, 2009). Unlike habitat generalists, which can change their habitat selection pattern over time (Latta & Faaborg, 2002), more specialised species are confined to a narrow spectrum of habitat types, potentially placing them under additional threats.

Habitat loss is a leading driver of population declines and local extinctions, particularly in the tropics, where species may be lost even before habitat associations are understood. This effect is even more critical for species that are naturally rare and endemic to restricted areas (Stattersfield et al., 1998; Pimm & Jenkins, 2010). Projections using human impacts on land use and climate change show that bird species with restricted distributions and specialised habitat requirements tend to lose 50% of their range size within 50 years, placing them under a double-jeopardy of extinction (Jetz, Wilcove & Dobson, 2007). To further compound the problem, restricted geographic distributions potentially place species at risk from heightened hunting pressure (Collar, Wege & Long, 1997).

The Cracidae (order Galliformes) are endemic to the Neotropics and one of the most threatened avian families (Collar, Wege & Long 1997; Brooks, Cancino & Pereira, 2006), with 24 species globally listed in some threat category (one EW, six CR, eight EN, nine VU; Table S1). As large terrestrial gamebirds, they are notably vulnerable to human disturbances through both habitat loss and selective hunting (Del Hoyo, 2017). A clear warning is provided by the Alagoas Curassow (*Mitu mitu*), which was driven to extinction in the wild by the combination of forest fragmentation and hunting (BirdLife International, 2016b). Although cracids serve important ecological roles as seed dispersers (Del Hoyo, 2017), the habitat relationships of most species have not been studied in detail because of small populations, restriction to inaccessible areas or cryptic behaviour (Delacour & Amadon, 2004).

The Wattled Curassow (*Crax globulosa*) is endemic to the Amazon basin where it occurs in river islands and floodplain forests that are seasonally flooded by white-water rivers in Brazil, Colombia, Ecuador, Peru, and Bolivia (Collar et al., 1992; Bennett, 2003; Haugaasen & Peres, 2008). Rapid population declines, to current estimates of just 250–1,000 birds, has placed *C. globulosa* on both the Brazilian and IUCN Red Lists as an Endangered Species (Hennessey, 1999; Bennett, 2000; MMA, 2014; BirdLife International, 2016a). As a range-restricted species under threat from both habitat loss and hunting, the Wattled Curassow is a prime example of a species where detailed information on its patterns of habitat selection could help inform conservation action. In particular, based on the importance of seasonal inundation events in defining access to water and food resources, as well as determining suitable nesting sites and exposure to predators, we expect distance to water to play a major role in their habitat selection (Collar et al., 1992). We used radio-telemetry to study the home range size and use of space of *C. globulosa*, quantifying its preferred small-scale habitat mosaic within Amazonian floodplain forests and investigating how preferences may affect future conservation prospects.

## Materials and Methods

### Study area

The study was conducted within the Uacari Sustainable Development Reserve (RDS Uacari; 632, 949 ha), Carauari municipality, Amazonas, Brazil. This state-managed reserve, established in 2005, comprises upland *terra firme* forest and seasonally flooded *várzea* forest along the margins of the white-water Juruá River. Nutrient-rich *várzea* soils, resulting from the deposition of pre-Andean alluvial sediments (Furch, 1997) during the annual flood pulse event (Junk, 1997), are characterised by a plant community with fast growth rates but lower wood density and forest stature than adjacent *terra firme* forests (Hawes et al., 2012). We conducted fieldwork in *várzea* floodplain forests along the Marari stream (5°45′05″S, 67°46′17″W), a tributary on the right bank of the Juruá River.

The Médio Juruá region has a wet, tropical climate with a mean annual rainfall of 3,679 mm (2008–2010; Bauana Field Station; 5°26′19″S, 67°17′12″W). Precipitation patterns (dry season: May–October; rainy season: November–April) correspond closely to the inundation period in *várzea* forests (terrestrial phase: July–December; aquatic phase: January–June) (Hawes & Peres, 2016), when the flood depth reaches up to 7.5 m (Junk et al., 2011). Mean monthly values of river discharge range from 135 to 1,407 m^3^/s (1973–2010; Porto do Gavião; Petrobrás S.A.), with a peak in January–May. Pronounced changes in habitat structure occur as a result of this marked seasonality, although flood depth and inundation period vary considerably with local topography (Hawes et al., 2012), influencing the distribution of both terrestrial and aquatic vertebrates (Hawes & Peres, 2014; Endo, Peres & Haugaasen, 2016).

### Home range size

We used VHF radio-telemetry to quantify the movement patterns and home range size of *C. globulosa*. We captured birds using 30 wooden traps known locally as ‘arapucas,’ which were set Monday–Friday and checked twice daily over 70 days (September–November 2014). To maximise capture rates, traps were set at sites where the species had been previously observed during intensive line-transect surveys (Leite, 2017). Each captured individual was blinded with a hood and its legs were tied to reduce stress, before drawing a blood sample from the brachial vein of the wing and fitting a VHF transmitter. This entire procedure, tested previously on simulated runs using domestic chickens, lasted no more than 15 min. All methods were approved by the ‘Comissão de Ética em Pesquisa no Uso de Animais’ at the Instituto Nacional de Pesquisas da Amazônia (024/2015) and fieldwork was authorised by the Ministério do Meio Ambiente/Instituto Chico Mendes de Conservação da Biodiversidade (40017-3).

We used backpack-type transmitters (150.00–150.99 MHz), manufactured to specification by Biotrack Ltd/Lotek Wireless, Inc. (Dorset, UK). Transmitters were black with an 18-month battery life, and weighed 46 g: equivalent to ~1.8% of the adult body mass for *C. globulosa* (~2,500 g) and well below the recommended limit of 3% (Kenward, 2001). An R-1000 model receiver (Communications Specialists, Inc., Orange, CA, US) and a three-element Yagi antenna were used to locate signals on a systematic basis between September 2014 and October 2015. Each tagged individual was monitored once daily, morning or afternoon, at least three times a week, for at least 12 months. When the radio-transmitter signal was located, we stealthily approached until direct visual observations were possible although all birds eventually fled as they were approached, with no clear indication of habituation to observers over time. We recorded flock size, position (ground or perched, in which case height above ground was recorded), and the straight-line distance to the nearest body of open water. The location where first observed and subsequent day-range locations (06:00–17:00 h) were recorded using a GPS (Garmin Map 60; Garmin, Olathe, KS, USA), starting with a different focal individual on each day to prevent any bias.

### Habitat selection

**Figure 1:**
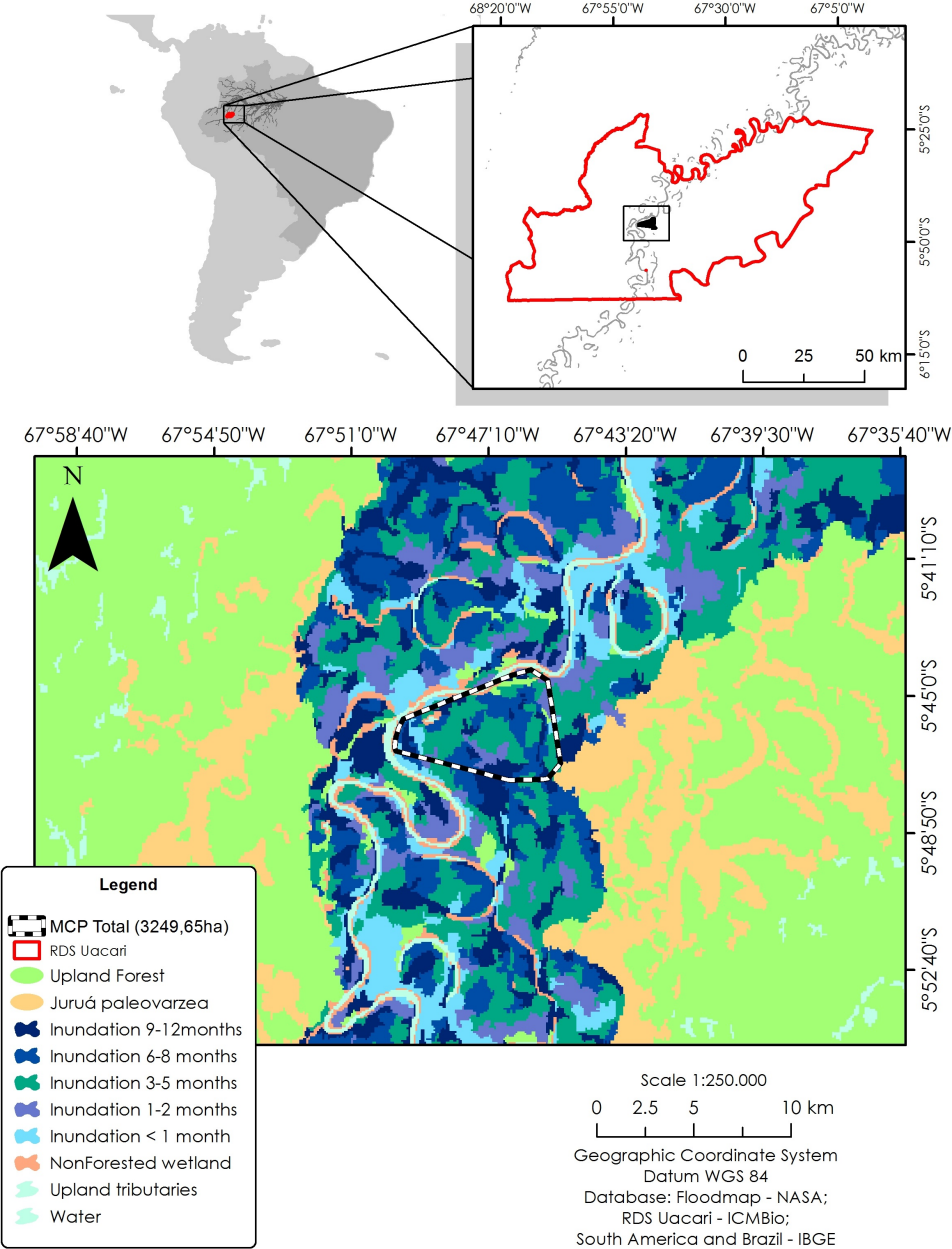
Study area in the Médio Juruá region of western Brazilian Amazonia, showing the forest and non-forest floodplain categories distinguished on the basis of ALOS ScanSAR images. Black–white line represents the 100% minimum convex polygon (MCP) for the three monitored individuals of *C. globulosa* across the study landscape.

To identify local habitat preferences, we used ranging data from a total of 579 locations. The study area was classified according to inundation period, using a flood map based on JAXA remote sensing data from the ALOS PALSAR system (see Hawes et al., 2012). Synthetic aperture radar (SAR) sensors are particularly useful to map flooded forests due to their ability to overcome problems of forest canopy cover, atmospheric and illumination conditions, and provide reliable measurements of flooding extent in wetland habitats (Arnesen et al., 2013). Based on a time series of 12 ScanSAR scenes from 2006 to 2011, a total of seven different forest categories were distinguished at a spatial resolution of 100 m: unflooded *terra firme* and *paleovárzea* forests, and seasonally inundated *várzea* forests with the following mean flooding periods: <1, one to two, three to five, six to eight, and nine to 12 months/year. In addition, we mapped all areas lacking vegetation cover, such as permanent open-water habitats (lakes and river channels), and sandy and alluvial sediment beaches of the main river and connecting levees. To calculate the area of each forest category, we used a 100% minimum convex polygon (MCP) as a representation of availability in relation to all floodplain habitat types (Fig. 1).

### Data analysis

#### Home range size

Location data were analysed using ArcGis 10.2.2 (ESRI (Environmental Systems Research Institute), 2014). Five different estimators were used to calculate home range size for comparison with other cracid studies: minimum convex polygon (95% MCP), fixed kernel (95% FK), Brownian bridge movement model (95% BBMM), and adaptive local convex hull (95% aLoCoH). We used a Kruskall–Wallis test to test for differences between home range sizes generated by the different estimators, using low sensitivity to extreme values and low amplitude as criteria for selecting the most representative estimators (Bernardo et al., 2011). We estimated home range sizes throughout the full year of monitoring and used a Wilcoxon signed-rank test to test for differences between dry and wet seasons.

#### Habitat selection

To assess habitat selection, we used the availability method, which compares the proportion of points sampled in a given habitat type with the proportion of expected points in the same habitat (Neu, Byers & Peek, 1974; Canavelli et al., 2003), with selection assumed to occur if habitat use is disproportionate to its overall local availability (Johnson, 1980; Alldredge & Griswold, 2006). We used a Chi-squared test to determine if habitat use was disproportionate to habitat availability (i.e. differences between observed and expected values), and a Wilcoxon test to detect any difference between wet and dry seasons (Thomas & Taylor, 2006), with the Bonferroni Confidence Interval used as necessary to account for multiple comparisons (Cherry, 1996). We included open water and non-vegetated areas even though they were not observed to be used, as their exclusion would not affect any analyses (Aebischer, Robertson & Kenward, 1993; Buskirk & Millspaugh, 2006).

We calculated distance to the nearest water source (oxbow lake, stream, river) for each location recorded, and used a Pearson correlation to test the hypothesis that the number of records would decline with increasing distance from open water. Finally, we used a Student’s *t*-test to test for a difference in this distance between seasons. All statistical analyses were conducted in R (R Development Core Team, 2016).

## Results

### Home range size

**Figure 2:**
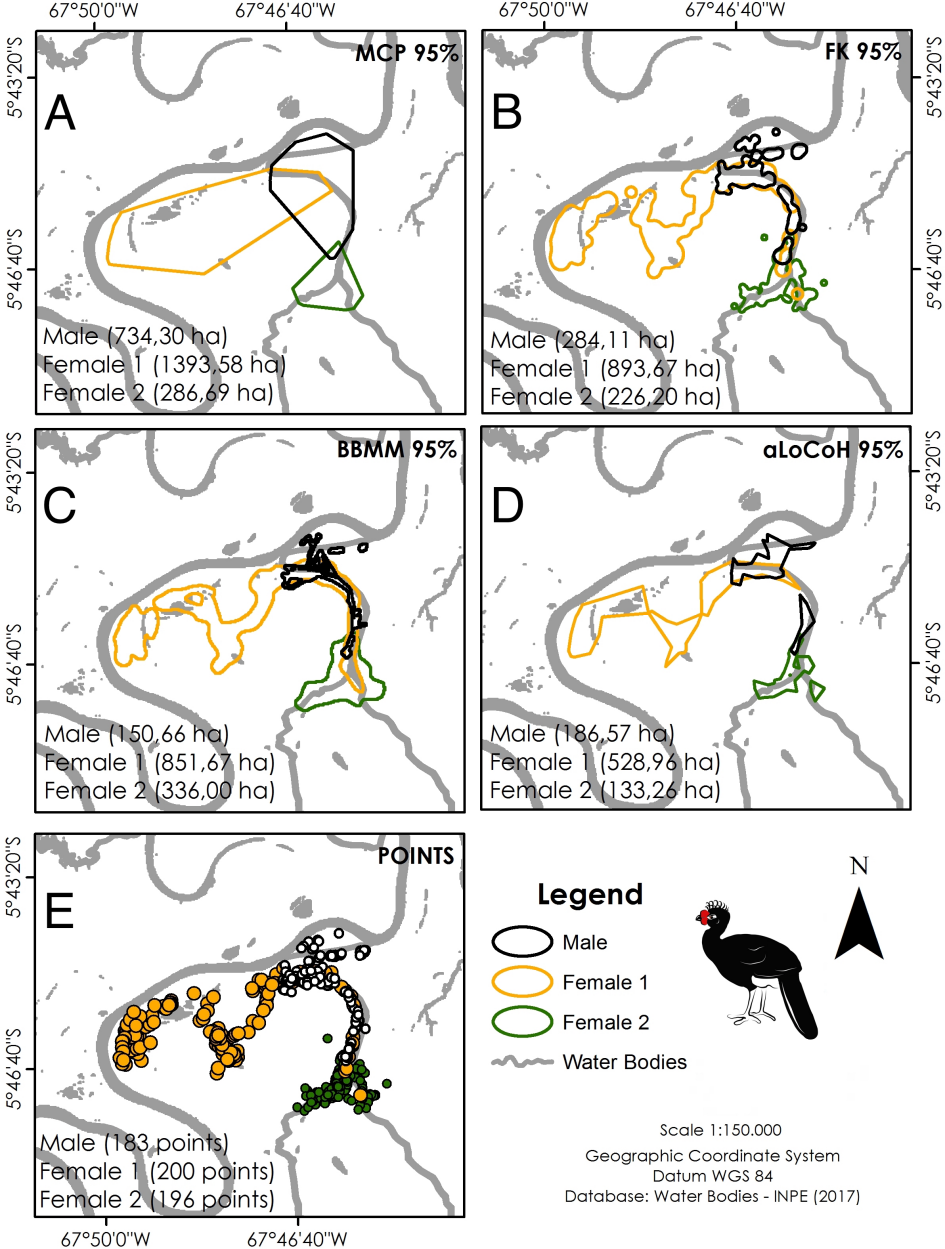
Home range size of the three monitored individuals of *C. globulosa* in the Médio Juruá, Amazonas, Brazil, using multiple estimators. (A) Minimum Convex Polygon (95% MCP). (B) Fixed Kernel (95% FK). (C) Brownian Bridge Movement Model (BBMM 95%). (D) Adaptive Local Convex Hull (aLoCoH 95%). (E) Locations of all individuals.

Our overall trapping effort (70 days × 30 traps = 2,100 trap-days) successfully captured only three *C. globulosa* (two females and one male; a fourth individual escaped the trap). All individuals remained in the same area of floodplain forest as their point of capture throughout the 12–13 month duration of monitoring. Our total of 579 positional records were well distributed across individuals and seasons (Table 1), with a mean of 192 points per individual that were used to calculate home range sizes (Table 2). The estimator with the lowest variance was aLoCoH, with a mean home range of 283 ± 214 ha (Fig. 2; Table 2). Average home range sizes in the wet (159 ± 62 ha) and dry seasons (146 ± 86 ha) were not significantly different (Wilcoxon: *V* = 2, *p* = 0.75) and there were no significant differences between the estimators used (Kruskal–Wallis: *H* = 3, *p* = 0.39). Female 1 had a larger home range size, in both dry (247 ha) and wet (227 ha) seasons, than either female 2 (100 ha dry and 105 ha wet) or the male (93 ha dry and 146 ha wet) (Fig. S1). In 51.4% of observations (*n* = 298) the tracked individual was not alone, with a flock size range of two to seven and mode of two (*n* = 142).

**Table 1:** Detail of radio-tracking effort (number of locations recorded) from three Wattled Curassows (*C. globulosa*) across the dry and wet seasons in the Médio Juruá region, Amazonas, Brazil.

Sex	Capture	Last day	*N* dry	*N* wet	*N* total
Female 1	15/09/2014	30/09/2015	112	88	200
Female 2	02/10/2014	01/10/2015	105	91	196
Male	18/10/2014	10/10/2015	90	93	183
Total			307	272	579

**Note:**

Dates show initial capture/fitting and the last day of monitoring.

**Table 2:** Home range size estimates (mean ± SD) for Wattled Curassow (*C. globulosa*) across the dry and wet seasons in the Médio Juruá region, Amazonas, Brazil.

Season	95% MCP^a^ (ha)	95% FK^b^ (ha)	95% BBMM^c^ (ha)	95% aLoCoH^d^ (ha)
Dry	537 ± 349	364 ± 143	344 ± 130	146 ± 86
Wet	482 ± 223	478 ± 421	296 ± 195	159 ± 62
All year	804 ± 556	468 ± 369	446 ± 363	283 ± 214

**Notes:**

a Minimum convex polygon.

b Fixed kernel.

c Brownian bridge movement model.

d Adaptive local convex hull.

### Habitat selection

**Figure 3:**
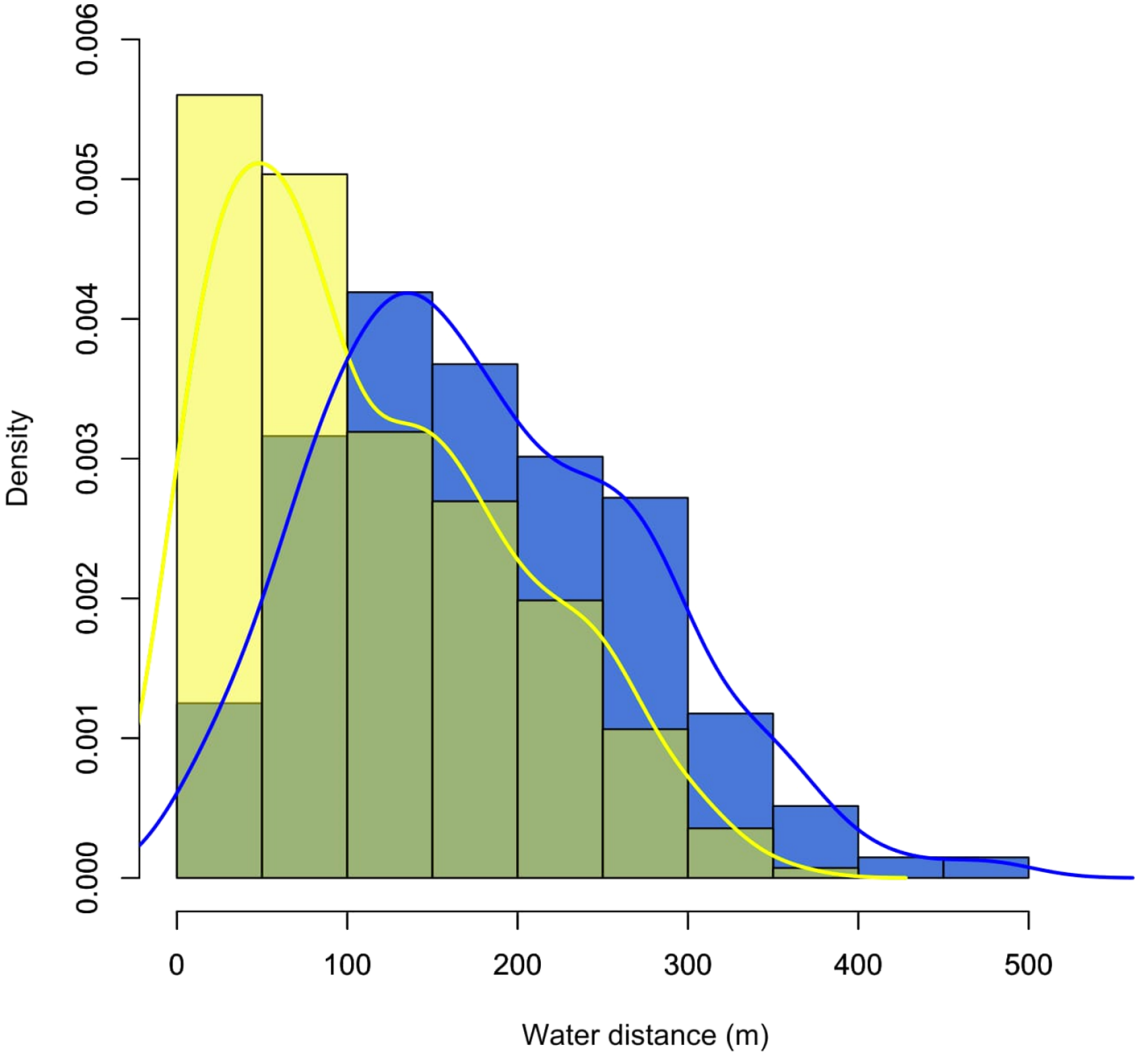
Density plot showing the distribution of records for the monitored individuals of *C. globulosa* in the Médio Juruá, Amazonas, Brazil, in relation to linear distances (m) to the nearest open waterbody. Yellow and blue bars and lines represent the dry and wet seasons, respectively.

The 100% MPC landscape macromosaic for all three Wattled Curassows monitored had an overall area of 3,249 ha and, although *C. globulosa* used almost all forest types, the contingency test indicated that observed use differed from the null expectation if forest types were selected randomly (Chi-square: *χ*^2^ = 52.98, gl = 7, *p* < 0.001). Wattled Curassows showed positive selection for areas of low-lying *várzea* forest that are flooded six to eight months/year, with double the number of expected locations observed if habitats were selected according to availability (Table 3). Other forest habitats had fewer observations than expected, no observations were made in open water or non-vegetated areas, and no *paleovárzea* habitat was present within the 100% MPC (Fig. 3). There was no difference in habitat selection between the dry and wet seasons (Wilcoxon: *V* = 8, *p* = 0.68).

**Table 3:** Bonferroni Confidence intervals for habitat selection by Wattled Curassow (*C. globulosa*) in the Médio Juruá region, Amazonas, Brazil, showing proportional use (%) for each habitat type used.

Habitat	Observed use (%)	Expected use (%)	Bonferroni’s intervals	Selection
Unflooded (*terra firme*)	0.02	0.02	0.006 < *p_i_* < 0.040	o
*Várzea* < one month	0.06	0.04	0.031 < *p_i_* < 0.086	o
*Várzea* one to two months	0.01	0.07	0.001 < *p_i_* < 0.027	−
*Várzea* three to five months	0.24	0.39	0.187 < *p_i_* < 0.286	−
*Várzea* six to eight months	0.56	0.28	0.504 < *p_i_* < 0.619	+
*Várzea* nine to 12 months	0.10	0.16	0.067 < *p_i_* < 0.137	−

**Note:**

Symbols represent the degree of habitat selection; (+): used more than expected; (−): used less than expected; (o): used as expected by chance.

Locations were consistently recorded near open water, with 83% of records (*n * = 521) less than 300 m from a stream or lake, and the farthest linear distance recorded from water being 930 m. The probability of encountering *C. globulosa* significantly decreased with increasing distance from any given waterbody (Pearson: *r* = −0.95, *p* < 0.001), and this preference for proximity to water was significantly higher (Student’s *t*-test: *t* = −8.82, df = 518.81, *p* < 0.001, Fig. 3) during the dry season (mean ± SD: 112 ± 83 m) compared to the wet season (184 ± 105 m). During the wet season, when the annual flood pulse effectively rendered all terrestrial habitat within low-lying floodplains unavailable, individuals were always recorded within the crowns of canopy trees (*n* = 272). In the dry season, we recorded individuals on the ground in 33.3% of observations (*n* = 102) and perched in trees in 66.7% of observations (*n* = 205).

## Discussion

### Home range size

On the basis of the smallest home range size of the four estimators we used, our three *C. globulosa* showed a larger mean home range size (283 ha) than other species of curassows studied to date with telemetry: *C. daubentoni* 149–197 ha (Bertsch & Barreto, 2008), *C. alector* 185 ha (Bernal & Mejía, 1995), *C. blumenbachi* 125 ha (Bernardo et al., 2011), and *M. salvini* 72–155 ha (Santamaría & Franco, 1994; Parra et al., 2001). The relatively low success of our trapping effort and subsequent small sample size, in terms of numbers of individuals, emphasises the practical difficulties in studying rare species in complex habitats. This issue should be considered as a potential caveat to our findings although the high-resolution of our tracking data is comparatively strong and most other curassow species have not been studied for a full year (Table 4). If we consider our home range sizes from each season separately, they are similar to those recorded for other curassow species (Table 2). The use of different habitats across seasons (areas nearer to water in the dry season), results in a larger home range overall, demonstrating the importance of data acquisition over the complete annual cycle. Climatic conditions during our study period (CPTEC/INPE, 2017) were consistent with long-term patterns (Hawes & Peres, 2016), indicating that our results should be representative of a typical year but tracking data across multiple years would be ideal to account for possible inter-annual variation.

**Table 4:** Comparison of Currasow studies showing sample effort, home range estimators used, home range size, and habitat use.

Species	Country	Sampling effort, months (N individual)	Analyses	Home range, ha	Habitat selection	Reference
*Crax globulosa*	Brazil	13 (3)	95% aLoCoH	283	*Varzea*, near to water	Leite et al. (this study)
			95% BBMM	446		
			95% FK	468		
			95% MCP	804		
*C. daubentoni*	Venezuela	3 (9)	95% FK	149	Gallery and dry forest	Bertsch & Barreto (2008)
			95% AK	197		
*C. alector*	Colombia	4 (4)	100% MCP	185	Riverine areas	Bernal & Mejía (1995)
*C. blumenbachi*	Brazil	25 (25)	95% NL	125	Riparian habitats	Bernardo et al. (2011)
*Mitu salvini*	Colombia	7 (4)	100% MCP	72–155	Flooded forest	Santamaría & Franco (1994); Parra et al. (2001)

**Note:**

AK, adaptative kernel; aLoCoH, adaptive local convex hull; BBMM, Brownian bridge movement model; FK, fixed kernel; MCP, minimum convex polygon; NL, neighbour linkage.

Results are also not easily comparable between different studies since analyses used different estimators and species exhibit different habitats and feeding habits. In studies of home range size in Galliformes, MCP, and Kernel estimators have been the most commonly used, although these potentially overestimate home range sizes and frequently associate tracked individuals with sites that they do not use (Bernardo, 2010). Had we considered only these estimators in our study, the home range size of *C. globulosa* would be estimated at 500–800 ha, an estimate well above the more conservative value we considered (aLoCoH: 283 ha), although differences between estimators were not significant.

Our finding that all tracked birds were consistently restricted to floodplain forests throughout the monitoring period, despite the availability of permanently unflooded *terra firme* forest only 3.5 km from the area monitored, and *de facto* unflooded *paleovárzea* forest only 100 m away, suggests that speculation of *C. globulosa* exhibiting lateral migration to adjacent forests on higher terrains during the wet season (BirdLife International, 2016a; Del Hoyo, Kirwan & Christie, 2017) may be mistaken. Instead, we report consistent horizontal and vertical movements within the mature *várzea* floodplain forest in response to the rising and falling floodwaters. One explanation for the persistence of *C. globulosa* in *várzea* forests during the wet season could be elevated fruit production at this time (Hawes & Peres, 2016), which provides a key food resource for the species (Ayres, 1993; Haugaasen & Peres, 2005), particularly from trees such as *Ficus maxima, Piranhea* sp., and *Ocotea* sp. (Leite, 2017). Therefore, although home ranges do not shift to unflooded *terra firme* during the wet season, seasonal movements to track fruit production within the spatially heterogeneous *várzea* floodplain habitat, coupled with the consistency in *C. globulosa* home range sizes across dry and wet seasons, may explain why we recorded a larger home range size than in other curassow species.

### Habitat selection

The strong preference we found for forest habitat near waterbodies and for forest experiencing flooding for six to eight months/year has also been observed for *C. globulosa* in Bolivia and Colombia, where the chance of detection decreases considerably at distances >250 m from water (Hill, Arañibar-Rojas & Macleod, 2008; Luna-Maira et al., 2013). These previous studies took place only in the dry season but, taken together with our more comprehensive sample over a complete annual cycle, these findings point strongly to a very narrow pattern of habitat selection by *C. globulosa*, thereby confirming its strict riverine or lacustrine floodplain distribution (Collar et al., 1992). Other curassow species, such as *C. blumenmachii* in south–east Brazil, also show a similar relationship with water (Collar et al., 1992; Bernardo, 2010).

Although the floodplain forest in our study region covers a large area, our findings indicate that *C. globulosa* shows a restricted preference for much narrower floodplain areas that are (i) flooded for prolonged periods, and (ii) near the banks of oxbow lakes, perennial streams or rivers. Movements away from these banks during the wet season may possibly reflect searching for food resources or an avoidance of hunters due to their greater exposure when in the forest canopy. Another important consideration is the breeding requirements of *C. globulosa*, with reproduction only occurring in the dry season when chicks are able to walk on the ground after hatching but with nests also located near open water, presumably to facilitate escape from predators (Leite et al., 2017). Dry season foraging may also contribute to the preference for areas close to lake or river banks, where there is a greater supply of other food resources such as fish, insects, and crustaceans (Delacour & Amadon, 2004), as well as mineral pebbles recorded in stomach contents that may serve to aid mechanical digestion within the gizzard (Leite, 2017), as proposed for other large-bodied cracids (Peres & Van Roosmalen, 1996).

### Double-jeopardy habitat use

The pattern of habitat selection confirmed by our study places the endangered *C. globulosa* in a clear case of habitat use double-jeopardy in relation to congruent spatial patterns of human-induced threats. This is consistent with other threatened species for which small to large scale spatial requirements converge with a high risk of direct or indirect human-induced mortality. For example, both marine and terrestrial megafauna are placed in double-jeopardy due to their large body size and the high commercial value of body parts such as shark fins and elephant ivory, with their market value increasing with species rarity (McClenachan, Cooper & Dulvy 2016). Within Neotropical birds, 78% of all endangered species have a distribution of less than 50,000 km^2^ and 57% are confined to wet forests, with habitat loss and intensive human exploitation as the main threats (Collar, Wege & Long, 1997).

*Crax globulosa* is firstly threatened by its restriction to seasonally flooded *várzea* forest which is one of the Amazonian ecosystems most influenced by human activities, with a 48% reduction in coverage over the last 35 years in the lower Amazon region alone (Renó et al., 2011; Junk & Piedade, 2005). *Várzea* forests are typically the first to be deforested in regions settled by riverine human populations, as they provide timber and easily accessible sites for the construction of new houses, as well as the planting of temporary monocultures such as cassava and grass pastures for domestic herbivores (Renó et al., 2011). Although floodplain forests cover an area of between 60,000 and 100,000 km^2^ of the Amazon (Junk, 1997), *C. globulosa* populations have an increasingly fragmented distribution within this area (BirdLife International, 2016a), elevating their vulnerability to further deforestation.

In addition to the general threat associated with a distribution restricted to *várzea* forests, remaining *C. globulosa* populations succumb to a second threat as a result of their faithful preference for habitats close to open water or those that remain flooded for six to eight months, since this habitat selection greatly increases their vulnerability to hunting. In our study region, this effectively reduced the total available area of *várzea* floodplain habitat to less than 8% of the overall *várzea*/*paleovárzea* land cover, severely reducing the effectively suitable habitat available for this species. Rivers are the primary transport network in the Amazon and people have inevitably settled along them, leading to the highest levels of human population density, and therefore demand for wild animal protein, occurring in areas closest to *várzea* forests. The higher levels of hunting associated with greater accessibility are responsible for driving declines in the abundance of a wide range of game species, including gamebirds (Peres & Lake, 2003). Finally, the most accessible areas within these flooded forests, such as riverbanks, streams, and lakes, are precisely the areas preferred by *C. globulosa*, with human threats further intensified during the dry season when hunters can easily explore *várzea* forests on foot.

This combination of factors increases the level of threat to the Wattled Curassow in the near future since, even at low densities, their habitat preferences place them at increased risk of extinction from habitat loss or hunting. There are three established forest reserves in our study region in the Médio Juruá, but these are classified as extractive or sustainable-use reserves, which do not prohibit hunting or logging. Hunting may have declined since the extensive commercial harvests of the 20th century skin trade (Antunes et al., 2016), but encounters with gamebirds such as *C. globulosa* are rarely passed up by opportunistic hunters (Michalski & Peres, 2017), leaving populations under continued threat despite relatively sustainable levels of local hunting pressure more generally (Abrahams, Peres & Costa, 2017). Avoiding the fate of the Alagoas Curassow, *M. mitu* (BirdLife International, 2016b), and improving the outlook for those species placed in similar double-jeopardy situations is unlikely to be achieved without conservation measures that account for their specific habitat requirements. For example, expanding a well-enforced protected area system, and ensuring that sufficiently large areas are protected from hunting, may be more successful if incorporating a zonal approach that targets protection of riparian habitat. Environmental education could also improve awareness of how the movement patterns of *C. globulosa*, and their subsequent role in seed dispersal, is of ecological importance in maintaining the dynamic floodplain ecosystem and other species that depend upon it.

## Conclusion

The small portions of floodplain habitat used by Wattled Curassows are both the most accessible to hunters and most vulnerable to deforestation. This restricted geographic and ecological distribution places them in a clear case of double jeopardy, with a much higher extinction risk at multiple spatial scales. Our results highlight the urgent need to consider habitat preferences within the conservation strategy for this and other floodplain forest species.

## Supplemental Information

10.7717/peerj.4617/supp-1 Supplemental Information 1 Summary of IUCN Red List status for all Cracidae genera, with all curassows in bold. EW = Extinct in the wild, CR = Critically Endangered, EN = Endangered, VU = Vulnerable, NT = Near Threatened, LC = Least Concern.

10.7717/peerj.4617/supp-2 Supplemental Information 2 Seasonal variation in home range size of the three monitored individuals of Crax globulosa in the Médio Juruá, Amazonas, Brazil. Solid and dashed lines represent the Adaptive Local Convex Hull (aLoCoH 95%) home ranges in wet and dry seasons, respectively.

## Additional Information and Declarations

### Field Study Permissions

The following information was supplied relating to field study approvals (i.e. approving body and any reference numbers): Fieldwork was authorised by the Ministério do Meio Ambiente/Instituto Chico Mendes de Conservação da Biodiversidade (project number: 40017-3).

### Data Availability

The following information was supplied regarding data availability: Movebank: ID number 385714539; https://www.movebank.org/panel_embedded_movebank_webapp.
