# Supplementary material for: Coarse- and fine-scale patterns of distribution and habitat selection places an Amazonian floodplain curassow in double jeopardy
Source: PeerJ. 2018 May 16;6:e4617. doi: 10.7717/peerj.4617 (PMC5960267; doi:10.7717/peerj.4617)
Supplement: Supplemental Information 1 — EW = Extinct in the wild, CR = Critically Endangered, EN = Endangered, VU = Vulnerable, NT = Near Threatened, LC = Least Concern. [file peerj-06-4617-s001.docx]

**Supplementary Material**

**Table S1.** Summary of IUCN Red List status for all Cracidae genera, with all curassows in bold. EW = Extinct in the wild, CR = Critically Endangered, EN = Endangered, VU = Vulnerable, NT = Near Threatened, LC = Least Concern.

| **Genus** | **EW** | **CR** | **EN** | **VU** | **NT** | **LC** | **Total** |
| --- | --- | --- | --- | --- | --- | --- | --- |
| *Aburria* |  |  |  |  | 1 |  | 1 |
| *Chamaepetes* |  |  |  |  | 1 | 1 | 2 |
| ***Crax*** |  | **2** | **3** | **3** |  |  | **8** |
| ***Mitu*** | **1** |  |  |  | **1** | **2** | **4** |
| ***Nothocrax*** |  |  |  |  |  | **1** | **1** |
| *Oreophasis* |  |  | 1 |  |  |  | 1 |
| *Ortalis* |  |  |  | 1 |  | 14 | 15 |
| ***Pauxi*** |  | **2** | **1** |  |  |  | **3** |
| *Penelope* |  | 1 | 2 | 4 |  | 8 | 15 |
| *Penelopina* |  |  |  | 1 |  |  | 1 |
| *Pipile* |  | 1 | 1 |  | 1 | 2 | 5 |
| **Total** | **1** | **6** | **8** | **9** | **4** | **28** | **56** |
