# Supplementary figures and images for: Coarse- and fine-scale patterns of distribution and habitat selection places an Amazonian floodplain curassow in double jeopardy
Source: PeerJ. 2018 May 16;6:e4617. doi: 10.7717/peerj.4617 (PMC5960267; doi:10.7717/peerj.4617)

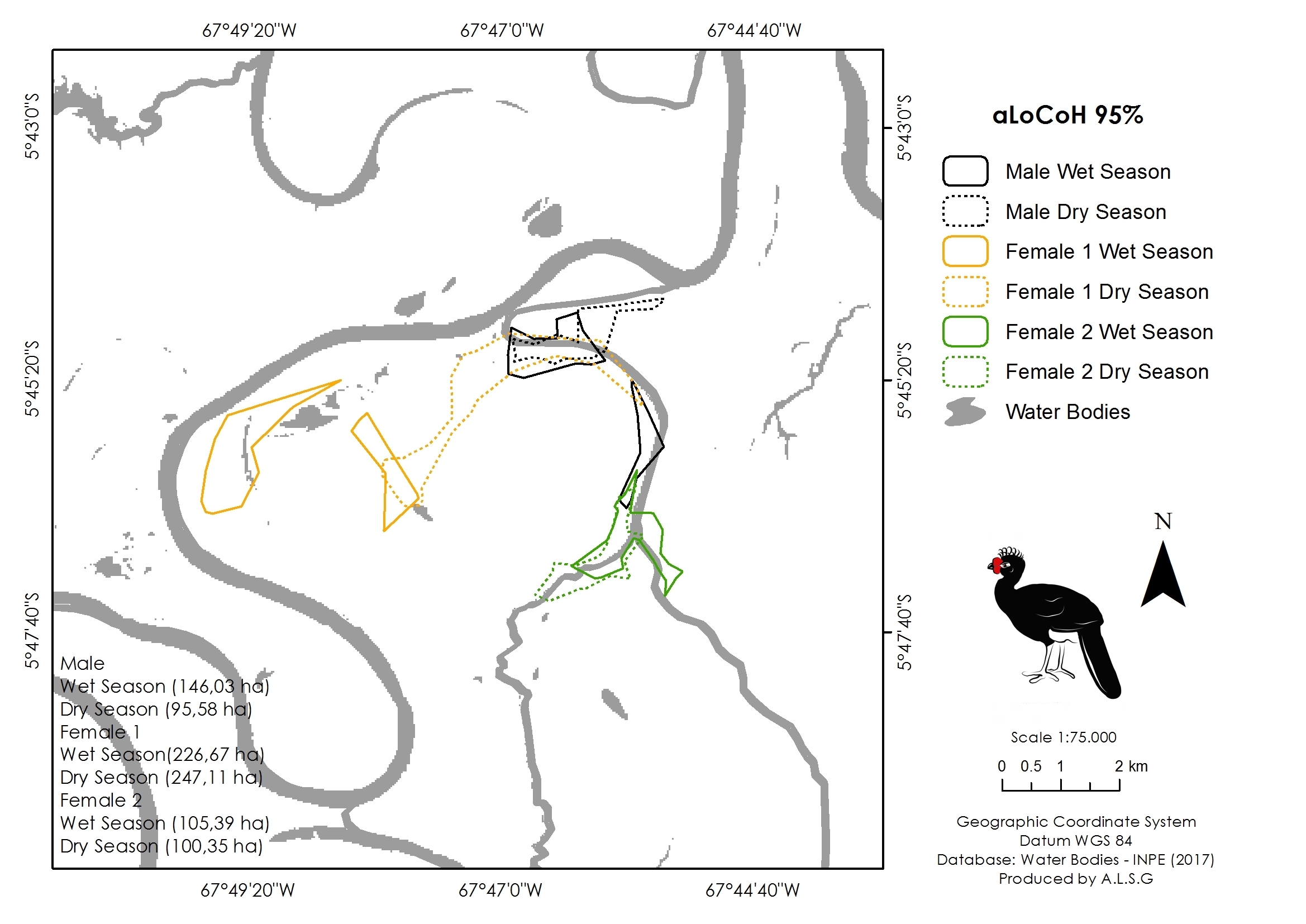

Supplement: Supplemental Information 2 — Solid and dashed lines represent the Adaptive Local Convex Hull (aLoCoH 95%) home ranges in wet and dry seasons, respectively. [file peerj-06-4617-s002.png]
